# Benzbromarone Inhibits Renal URAT1 and Attenuates Renal Damage in Streptozotocin-Induced Diabetic Rats, Independent of Its Uricosuric Effects

**DOI:** 10.1155/jdr/9934190

**Published:** 2025-11-25

**Authors:** Chuping Chen, Ping Zhu, Rongshao Tan, Yan Liu, Jianmin Ran

**Affiliations:** ^1^Endocrinology and Metabolism Department, Guangzhou Red Cross Hospital Affiliated to Jinan University, Guangzhou, China; ^2^Institute of Diseases-Oriented Nutrition Research, Guangzhou Red Cross Hospital Affiliated to Jinan University, Guangzhou, China

**Keywords:** benzbromarone, diabetes, GLUT9, rats, renal damage, URAT1

## Abstract

**Background:**

Hyperuricemia remains a critical risk factor for diabetic kidney disease (DKD) currently. Recent studies have confirmed that allopurinol, an inhibitor of xanthine oxidase (XO), does not have any beneficial effects on DKD. However, it is still unclear how uricosurics, such as benzbromarone (BZ), affect the progression of DKD.

**Methods:**

STZ-induced diabetic and control rats were treated with BZ for 8 weeks. Blood samples were collected to measure fasting blood glucose (FBG), serum uric acid (SUA), serum creatinine (SCr), and blood urea nitrogen (BUN). Simultaneously, urinary samples were tested, and the daily urinary amounts of albumin (UAE), uric acid (UUA), creatinine (UCr), urea nitrogen (UUN), were calculated. Gene expressions of XO, urate transporter 1 (URAT1), and glucose transporter 9 (GLUT9) in the kidney, as well as XO, uricase, and GLUT9 in the liver, were detected.

**Results:**

(1) Compared with normal rats, diabetic rats exhibited significant increases in FBG, BUN, UUN, UAE, UUA, while SUA was significantly decreased. BZ significantly decreased UAE and increased SUA over 8 weeks in diabetic rats. (2) Diabetic rats developed noticeable hyaline degeneration, and a slight decrease in mean glomerular area. BZ treatment significantly attenuated tubular damage in diabetic rats without affecting glomerular morphology. (3) In the kidney, gene expression of XO was increased, while URAT1 and GLUT9 were unchanged in diabetic rats. BZ treatment had no effect on GLUT9 and XO gene expression but significantly inhibited URAT1 expression in diabetic rats. (4) In the liver, gene expression of XO, uricase, and GLUT9 did not differ between diabetic and normal rats. BZ treatment significantly inhibited GLUT9 expression but had no effect on XO and uricase expression in diabetic rats

**Conclusions:**

BZ treatment significantly protects against renal damage in STZ-induced diabetic rats independent of its uricosuric effects, possibly because of its inhibition of renal URAT1.

## 1. Background

Diabetic kidney disease (DKD) is the leading cause of chronic kidney disease (CKD) and end-stage renal disease (ESRD) worldwide [1, 2]. It poses a heavy health burden on individuals and healthcare systems [2] and is closely associated with higher risks of cardiovascular events and even mortality [3, 4]. In recent years, despite the identification of several key pathways, including the renin–angiotensin system (RAS), inflammation and oxidative stress, fibrosis and extracellular matrix accumulation, and epigenetic modifications, effective treatments to halt or reverse DKD remain limited [5, 6].

Hyperuricemia has been established as a critical risk factor for DKD in various recent cross-sectional and cohort studies [7–9]. However, therapeutics targeting uric acid lowering have yielded controversial results. Allopurinol, a traditionally specific inhibitor of xanthine oxidase (XO), failed to improve kidney outcomes among patients with Type 1 diabetes accompanied by early-to-moderate DKD in the PERL study [10]. Among patients with Type 2 diabetes mellitus (T2DM), diverse outcomes related to the attenuation of estimated glomerular filtration rate (eGFR) and microalbuminuria after allopurinol treatment have been shown [11–13], despite the decrease in serum uric acid (SUA) levels observed in these studies. As a result, most researchers and clinicians now share the common opinion that uric acid-lowering therapy (ULT) or these agents themselves contribute limited effectiveness to the prognosis of DKD [14].

Benzbromarone (BZ) is a uricosuric agent that promotes uric acid (UA) excretion by inhibiting urate transporter 1 (URAT1) and glucose transporter 9 (GLUT9), which are localized to the apical and basolateral membranes of proximal tubular cells, respectively [15]. In a cohort study involving 874 patients with different stages of CKD, Chou et al. found that BZ is more effective in reducing the risk of progression to ESRD over a 13-year follow-up when compared with allopurinol and febuxostat, two XO inhibitors [16]. In another perspective study with a 12-month follow-up [17], patients with CKD with hyperuricemia and an eGFR of 20–60 mL/min/1.73 m^2^ were treated with BZ and febuxostat, respectively. Both ULT agents were able to maintain renal function and potentially improve anemia. However, several other studies [18] using BZ in patients with CKD did not yield promising outcomes. In a rat model with obesity and hypertension induced by a high-fructose diet, Zhang et al. found that the ULT by treatment with either allopurinol or BZ attenuated weight gain and reduced blood pressure [19]. To date, there have been no targeted studies on BZ treatment in patients with DKD, despite the agent still being widely prescribed in Asian countries and other regions [20]. Therefore, we conducted this pilot study in streptozotocin (STZ)-induced diabetic rats with the aim of exploring the effect of BZ on renal damage and the possible underlying mechanisms.

## 2. Methods

### 2.1. Animal Preparation

**Figure 1:**
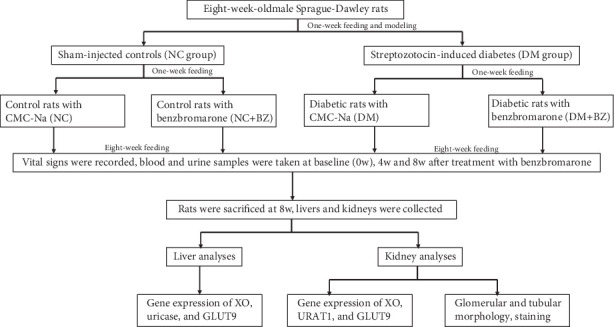
Schematical protocol of the overall experiment. NC: normal control rats, NC + BZ: normal control rats with benzbromarone treatment, DM: diabetic rats, DM + BZ: diabetic rats with benzbromarone treatment, BZ: benzbromarone, XO: xanthine oxidase, GLUT9: glucose transporter 9, URAT1: urate transporter 1.

The overall protocol of the study is schematically shown in Figure 1. Eight-week-old male Sprague-Dawley rats (Guangdong Medical Laboratory Animal Center, China) weighing 250–280 g were used. Forty-eight rats were housed two rats per cage and fed with standard rat chow for 2 weeks. Diabetes was induced by a single intraperitoneal injection of 1% STZ (MP Biomedicals, CA, United States) at a dose of 65 mg/kg, and those with random blood glucose levels over 16.7 mmol/L in three different times were selected for the following experiments. Rats injected with the same volume of citrate buffer served as controls.

### 2.2. BZ Treatment and Animal Experiments

One week after modeling, rats were further randomized into four groups, each containing 12 rats: diabetic rats with benzbromarone (DM + BZ), diabetic rats (DM), control rats with benzbromarone (NC + BZ), and control rats (NC). BZ (Shanghai Macklin Biochemical Technology Co., Ltd., China) was dissolved in 0.5% sodium carboxymethyl cellulose (CMC-Na) to prepare a 1.5 mg/mL solution. Rats in the DM + BZ and NC + BZ groups received BZ by oral gavage at 15 mg/kg/day for 8 weeks, whereas those in the DM and NC groups were only given an equivalent volume of 0.5% CMC-Na. The basal state (0 W) of the experiment was defined as the start of BZ treatment, which began approximately 2 weeks after the STZ injection used for modeling.

Throughout the entire experiment, all rats had ad libitum access to food and water, and the room lighting followed a 12-h light-dark cycle. Rats were kept fasting for 12 h before the experiment. Vital signs, including mean arterial pressure (MAP) and heart rate (HR), were recorded in completely conscious rats using indirect tail-cuff equipment (LE5002, Harvard Apparatus, United States). Blood samples were collected at baseline, 4, and 8 weeks after BZ treatment for biochemical assays. Daily urine was collected and quantified before the experimental day while rats were housed in special metabolic cages. Rats were euthanized by exsanguination after being anesthetized with 3% pentobarbital sodium (1.5 mL/kg body weight) following 8 weeks of BZ treatment. Kidneys and livers were then collected for histological examination and gene expression analysis. The study was conducted in accordance with the National Institutes of Health Guide for the Care and Use of Laboratory Animals (Eighth edition, 2011) and ARRIVE guidelines 2.0. The experimental protocol was approved by the Ethics Committee of Guangdong Medical Laboratory Animal Center (No. B201708-19).

### 2.3. Biochemical Assays and Calculations

Blood samples for the detection of fasting blood glucose (FBG), SUA, serum creatinine (SCr), and blood urea nitrogen (BUN) were measured using corresponding commercial kits on an automatic biochemical machine (HITACHI, Japan). On the same system, urinary samples were tested for the concentration of UA, creatinine, and urea nitrogen. The urinary albumin concentration was measured using the Bradford method. The daily urinary amounts of UA (UUA), creatinine (UCr), urea nitrogen (UUN), and albumin excretion (UAE) were calculated by multiplying each concentration by the daily urine volume.

The renal clearance rate of creatinine (Ccr) and UA (Cua) was estimated according to the following formulas, respectively: Ccr (mL/min) = [UCr (*μ*mol/L) × urine volume (mL)/SCr (*μ*mol/L)] / 1440 min, Cua (mL/min) = [UUa (*μ*mol/L) × urine volume (mL)/SUA (*μ*mol/L)] / 1440 min. UCr and UUa represent urinary concentrations of creatinine and UA, respectively. Fractional excretion of urate (FEUA), which represents the percentage of filtered UA excreted in the final urine, was calculated as (Cua/Ccr) × 100%. FEUA is a common and specific index for indicating the tubular reuptake of urate normalized to its filtered load both in human and murine models.

### 2.4. Renal Histological Evaluation

Kidneys from all rats were harvested, fixed in 4% paraformaldehyde, embedded in paraffin, and sectioned at 2 *μ*m. For morphological assessment, the sections were stained using the routine Hematoxylin-Eosin (H&E) staining and PAS methods. All slides were digitized and processed using the specific computer system (BX41, Olympus, Japan; and Mshot, China). The degree of tubular hyaline degeneration was semiquantitatively scored as follows: 0 for absent, 1 for mild (< 25% of total observed areas), 2 for moderate (25%–50% of total observed areas), and 3 for severe (> 50% of total observed areas) [21]. The glomerular area was outlined along the capillary loop, and the mean glomerular area (MGA) was determined from the average of 15 glomeruli.

### 2.5. Gene Expression

The renal gene expression of XO, URAT1, GLUT9, and hepatic gene expression of XO, Uricase, and GLUT9 were determined using real-time PCR. Frozen tissues were homogenized, and total RNA was extracted using a TRIzol kit (Takara, Japan). Real-time PCR was performed using SYBR Green PCR Master Mix reagent kits after reverse transcription to cDNA, with *β*-actin used as the housekeeping gene. As described previously [22], RT-qPCR was performed with an initial denaturation at 94°C for 5 min, followed by 40 cycles of 95°C for 5 s, 65°C for 34 s, and 72°C for 30 s. Primer sequences are provided in Table 1, and all oligonucleotides were synthesized by Sangon Biotech (Shanghai, China).

### 2.6. Statistical Analysis

The data are expressed as mean ± SD. One-way analysis of variance (ANOVA) was used to compare the differences in means. The least significant difference (LSD) test was then employed for comparisons between two groups. A significance level of *p* < 0.05 was considered statistically significant.

## 3. Results

### 3.1. General Characteristics

**Figure 2:**
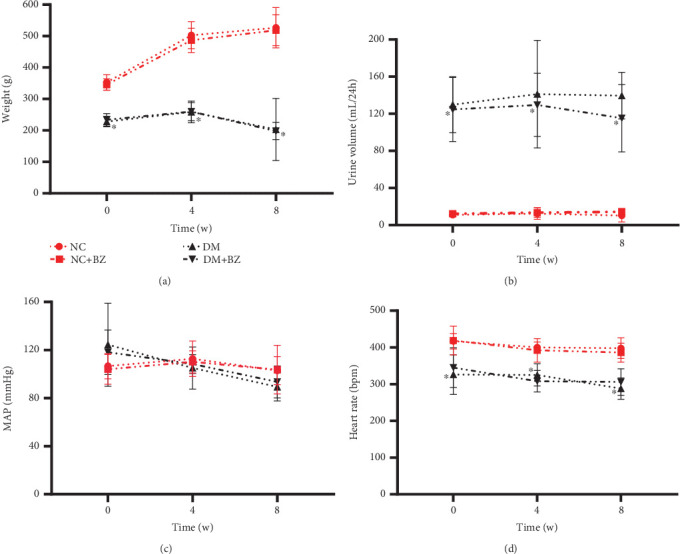
Time course of vital signs among different rat groups. Mean arterial pressure and heart rate were recorded using an indirect tail-cuff method (*N* = 12 for each group). Each rat group is represented by a different line with symbol style, as illustrated in panel (a) above. Panel (a) for body weight, (b) for urine volume, (c) for MAP, and (d) for heart rate, respectively. NC: normal control rats, NC + BZ: normal control rats with benzbromarone treatment, DM: diabetic rats, DM + BZ: diabetic rats with benzbromarone treatment, BZ: benzbromarone, MAP: mean arterial pressure. ⁣^∗^*p* < 0.05 compared to the NC group.

As depicted in Figure 2, diabetic rats consistently exhibited significantly lower body weight compared with the control rats throughout the entire experiment (*p* < 0.05). Treatment with BZ did not significantly impact body weight in both groups of rats (Figure 2a). The daily urine volume of diabetic rats was significantly higher than that of the control rats (*p* < 0.05). BZ treatment slightly reduced the daily urine volume in diabetic rats, but this reduction was not statistically significant (Figure 2b). MAP (Figure 2c) was similar between diabetic and control rats, while diabetic rats displayed a lower HR (Figure 2d) than normal rats (*p* < 0.05) throughout the entire experiment. BZ treatment did not affect MAP or HR in either diabetic or normal rats (Figure 2c,d).

### 3.2. Blood Biochemistry

**Figure 3:**
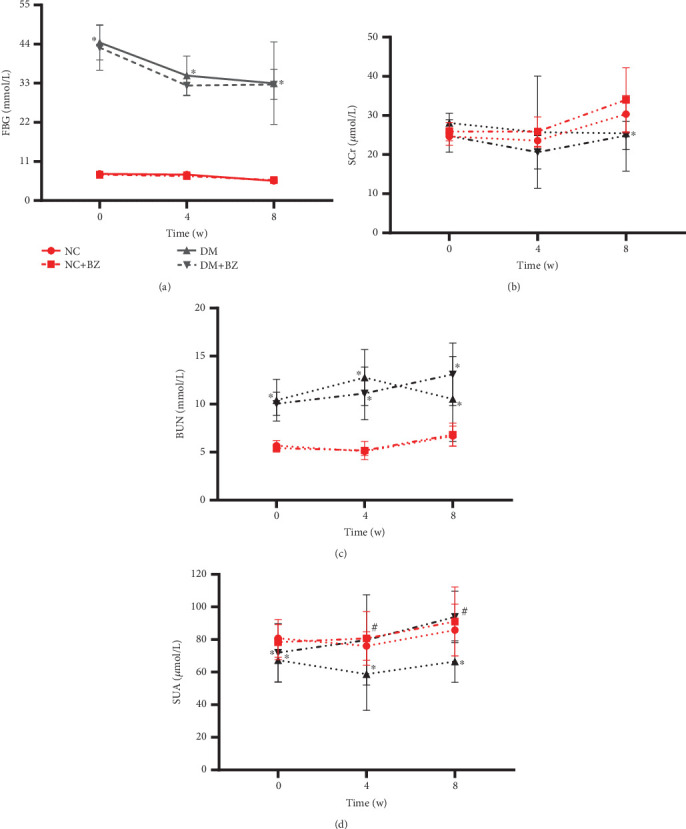
Time course of serum biochemical parameters among different rat groups (*N* = 12 for each group). Each rat group is represented by a different line with symbol style, as illustrated in panel (a) above. Panel (a) for FBG, (b) for SCr, (c) for BUN, and (d) for SUA, respectively. NC: normal control rats, NC + BZ: normal control rats with benzbromarone treatment, DM: diabetic rats, DM + BZ: diabetic rats with benzbromarone treatment, BZ: benzbromarone, FBG: fasting blood glucose, SCr: serum creatinine, BUN: blood urea nitrogen, SUA: Serum uric acid. ⁣^∗^*p* < 0.05 compared to the NC group, ^#^*p* < 0.05 compared to the DM group.

Blood glucose levels remained elevated in diabetic rats throughout the experiment (FBG was 5.56 ± 0.54 and 33.00 ± 11.61 mmol/L for the NC and DM group, respectively, even at Week 8, *N* = 12 for each group, *p* < 0.05), and BZ treatment had no effect on FBG in both groups of rats (Figure 3a). Regarding renal function parameters, Scr was comparable between diabetic and control rats, while BUN was significantly increased in diabetic rats throughout the entire experiment (*p* < 0.05). BZ treatment did not cause any significant changes in Scr and BUN in either group of rats (Figure 3c,d). Interestingly, SUA levels were significantly decreased in diabetic rats compared with control rats (*p* < 0.05), and BZ treatment significantly increased SUA levels in diabetic rats (*p* < 0.05), while it remained unchanged in control rats (Figure 3d).

### 3.3. Urine Biochemistry and Calculated Parameters

**Figure 4:**
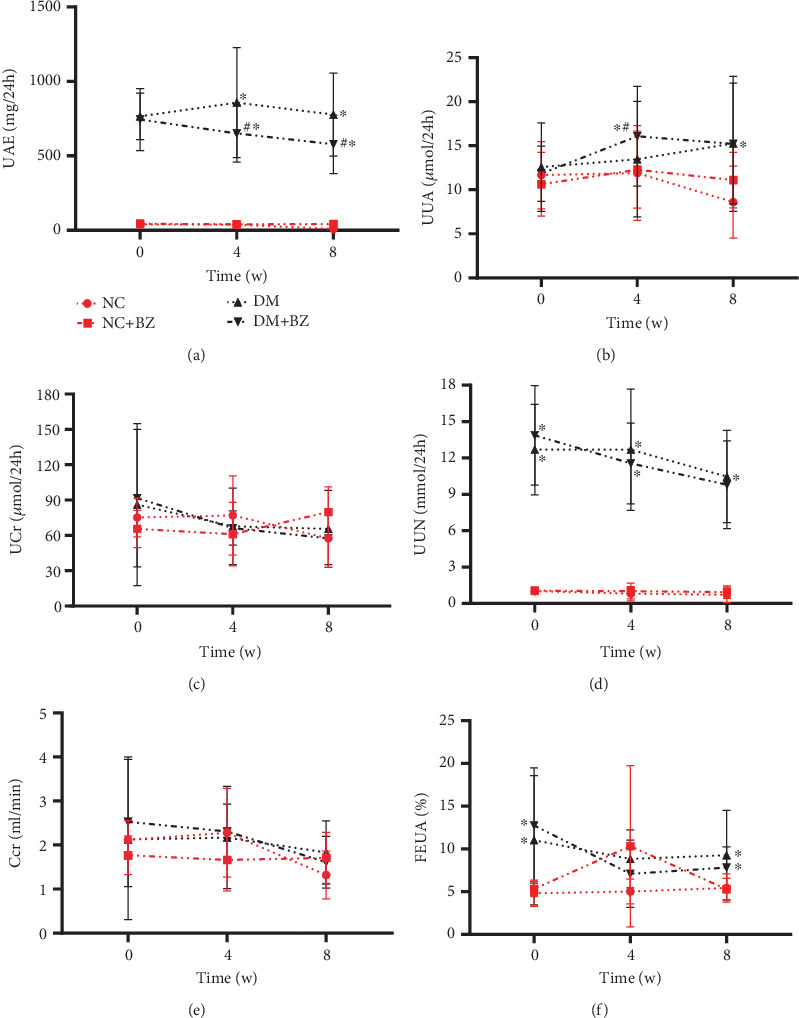
Time course of urinary biochemical and calculated parameters among different rat groups (*N* = 12 for each group). Each rat group is represented by a different line with symbol style, as illustrated in panel (a) above. Panel (a) for UAE, (b) for UUA, (c) for UCr, (d) for UUN, (e) for Ccr, and (f) for FEUA, respectively. NC: normal control rats, NC + BZ: normal control rats with benzbromarone treatment, DM: diabetic rats, DM + BZ: diabetic rats with benzbromarone treatment, BZ: benzbromarone, UAE: urinary albumin excretion per day, UUA: urinary amounts of uric acid per day, UCr: urinary amounts of creatinine per day, UUN: urinary amounts of urea nitrogen per day, Ccr: clearance rate of creatinine, FEUA: fraction excretion of urate. ⁣^∗^*p* < 0.05 compared to the NC group, ^#^*p* < 0.05 compared to the DM group.

As depicted in Figure 4, UAE was significantly increased in diabetic rats compared with control rats after modeling (765.26 ± 155.76 vs. 39.93 ± 14.96* μ*mol/L for the DM and NC group, respectively, *N* = 12 for each group, *p* < 0.05), and BZ treatment notably decreased it at the 4th and 8th week (857.47 ± 369.57 vs. 653.60 ± 193.07* μ*mol/L at the 4th week, 776.49 ± 278.10 vs. 577.66 ± 195.89* μ*mol/L at the 8th week, for the DM and DM + BZ group, respectively, *N* = 12 for each group, both *p* < 0.05). UUA levels were also increased in diabetic rats, with a slight further increase at the 4th week after BZ treatment (14.32 ± 6.18* μ*mol/24 h vs. 16.08 ± 5.67* μ*mol/24 h for the DM and DM + BZ group, respectively, *N* = 12 for each group, *p* < 0.05), but were comparable at the 8th week (Figure 4). Ucr level was similar between diabetic and control rats throughout the experiment, and BZ treatment did not alter the Ucr level in diabetic rats (Figure 4). However, UUN in diabetic rats was significantly higher than that in control rats (*p* < 0.05) and remained unchanged after BZ treatment in these rats (Figure 4). As shown in Figure 4, Ccr remained comparable among the four groups throughout the different experimental stages, while FEUA was significantly higher in diabetic rats at Week 0 and Week 8 (11.03% ± 7.55% vs. 4.82% ± 1.53% at Week 0, 9.27% ± 5.24% vs. 5.44% ± 1.66% at Week 8, for the DM and control group, respectively, *N* = 12 for each group, both *p* < 0.05) (Figure 4). BZ treatment slightly increased FEUA at Week 0, but without statistical significance, while FEUA remained comparable at both Week 4 and Week 8 (Figure 4).

### 3.4. Kidney Histology

**Figure 5:**
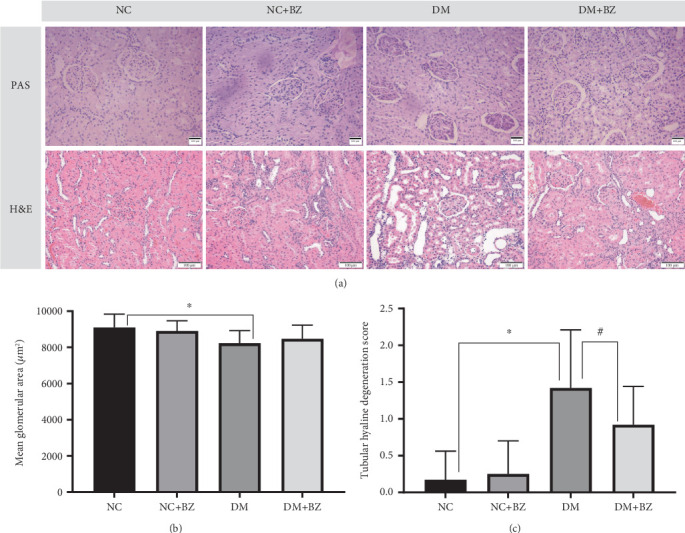
Renal morphology alterations among different rat groups (*N* = 12 for each group). Renal slices were stained in two different methods including PAS and Hematoxylin–Eosin. MGA and tubular degeneration score were measured and calculated. Panel (a) displays representative glomerular and tubular images in two staining methods from four groups, respectively. Panels (b and (c) show the semiquantitative MGA and tubular degeneration score among the different rat groups. H&E: hematoxylin–eosin staining method. MGA: mean glomerular area NC: normal control rats, NC + BZ: normal control rats with benzbromarone treatment, DM: diabetic rats, DM + BZ: diabetic rats with benzbromarone treatment, BZ: benzbromarone. ⁣^∗^*p* < 0.05 compared with the NC group, ^#^*p* < 0.05 compared with the DM group.

Representative images of kidneys with PAS and H&E staining are shown in Figure 5a. Compared with the control rats, diabetic rats displayed smaller glomeruli and mild tubular hyaline degeneration. BZ treatment did not induce significant morphological changes in the glomeruli of either the control rats or the diabetic rats. As shown in Figure 5b, the semiquantitative MGA in diabetic rats was slightly lower than that in control rats (8222.96 ± 699.75 vs. 9093.73 ± 739.81* μ*m^2^ for the DM and NC group, respectively, *N* = 12 for each group, *p* < 0.05), but BZ treatment did not affect it in either group. However, as depicted in Figure 5c, the tubular degeneration score in diabetic rats was significantly higher than that in control rats (1.42 ± 0.79 vs. 0.17 ± 0.39 for the DM and NC group, respectively, *N* = 12 for each group, *p* < 0.05). BZ treatment significantly reduced the score (1.42 ± 0.79 vs. 0.92 ± 0.52 for the DM and DM + BZ group, respectively, *N* = 12 for each group, *p* < 0.05) in diabetic rats but did not change it in the control rats (Figure 5c).

### 3.5. Gene Expression in Kidney and Liver

**Figure 6:**
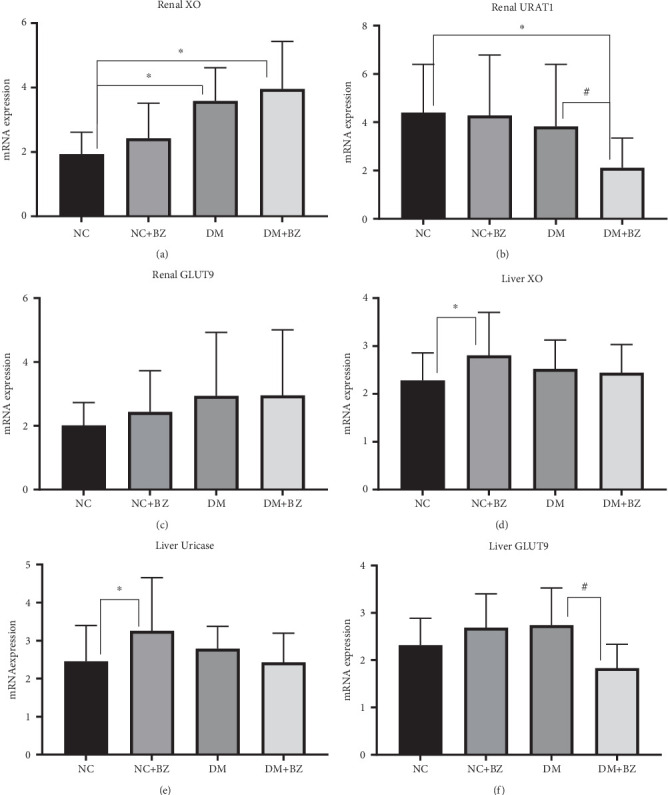
Renal and hepatic gene expressions among different rat groups (*N* = 12 for each group). Panel (a) for renal XO, (b) for renal URAT1, (c) for renal GLUT9, (d) for liver XO, (e) for liver uricase, and (f) for liver GLUT9, respectively. NC: normal control rats, NC + BZ: normal control rats with benzbromarone treatment, DM: diabetic rats, DM + BZ: diabetic rats with benzbromarone treatment, BZ: benzbromarone. ⁣^∗^*p* < 0.05 compared to the NC group, ^#^*p* < 0.05 compared to the DM group.

Renal XO gene expression was significantly higher in diabetic rats compared with control rats (*p* < 0.05), and BZ treatment did not significantly alter expression levels in either group of rats (Figure 6a). As shown in Figure 6b, renal URAT1 gene expression was comparable among the NC, NC + BZ, and DM groups but was significantly inhibited after BZ treatment (3.825 ± 2.573 vs. 2.109 ± 1.235 for the DM and DM + BZ group, respectively, *N* = 12 for each group, *p* < 0.05). Renal GLUT9 gene expression was similar among rats in all four groups, with and without BZ treatment (Figure 6c).

As shown in Figure 6d,e, hepatic XO and uricase gene expression were not significantly different in diabetic rats compared with control rats. However, in control rats, BZ treatment slightly increased the expression of both genes (*p* < 0.05), rather than in diabetic rats. Hepatic GLUT9 gene expression was similar among the NC, NC + BZ, and DM groups, while BZ treatment significantly inhibited it in diabetic rats (2.739 ± 0.785 vs. 1.830 ± 0.505 for the DM and DM + BZ group, respectively, *N* = 12 for each group, *p* < 0.05), as depicted in Figure 6f.

## 4. Discussion

In the present study, the most interesting finding is that BZ attenuates urinary albumin excretion in STZ-induced diabetic rats without lowering blood glucose levels or SUA. In addition, BUN and UUN were significantly elevated in diabetic rats, which may have resulted from increased hyperphagia and protein intake for turnover to urea. Conversely, SCr was reduced at Week 8, possibly as a consequence of the marked reduction in body weight and muscle mass in these animals. Importantly, BZ treatment had no effect on either parameter in diabetic or control rats. Likely, despite significant improvements in tubular damage after BZ treatment in diabetic rats, no visible renal glomerular repair has been observed. Similar renal damage, including tubular hyaline degeneration and decreased MGA, has been observed in our previous studies using the same rat model treated with a low protein diet (LPD) and febuxostat, respectively [23, 24]. Twelve weeks of treatment with LPD effectively attenuated tubular hyaline score [23], but 8 weeks of treatment with febuxostat, a specific XO inhibitor, did not significantly influence renal morphology [24]. Therefore, we can hypothesize that extended therapy with BZ may improve renal structural damage in addition to its effect of reducing urinary albumin excretion. Proteinuria and albuminuria have long been established as early markers of renal function deterioration [25, 26], and therapeutics targeting proteinuria or albuminuria have shown remarkable effectiveness in slowing the progression to ESRD in patients with DKD and CKD [27–29]. In this study, diabetic rats exhibited increased renal UA and urea nitrogen excretion, while creatinine excretion remained unchanged. After 4 weeks of BZ treatment, renal UA excretion increased further, suggesting that BZ has a mild uricosuric effect. However, since the FEUA was significantly higher in diabetic rats at both Week 0 and Week 8, and BZ treatment did not alter them significantly, we could not attribute this uricosuric effect to the inhibitory effects of BZ on the urate transporter.

To date, there have been few clinical studies investigating the prognosis of DKD with uricosuric treatment, although several studies [15, 16] have shown that BZ may improve renal function outcomes in patients with CKD, many of whom do not have diabetes. Dotinurad, a novel uricosuric agent that specifically inhibits renal URAT1 but not GLUT9 [30], has also been shown to be effective in slowing the decline of eGFR in a retrospective study involving patients with CKD [31]. In another retrospective study, which included 59.3% of participants with Type 2 diabetes, dotinurad also demonstrated significant renal protection [32]. Yanai et al. [33] reported a case of a patient with Type 2 diabetes and CKD (stage G4) who showed improvement in eGFR and albumin–creatinine ratio (ACR) after dotinurad treatment, in addition to multidisciplinary therapies. Moreover, a single-arm, open-label, prospective study [34] is currently underway, aiming to observe the effects of URAT1 inhibitor on parameters related to renal injury in patients with Type 2 DKD and hyperuricemia who switched from febuxostat to dotinurad. However, since BZ remains an effective and safe agent in most Asian countries, our findings in STZ-induced diabetic rats support the notion that URAT1 inhibitors may be a novel target for DKD treatment.

The possible mechanisms underlying renal protection of BZ in diabetic rats were also investigated. BZ promotes tubular UA excretion by inhibiting renal URAT1 and GLUT9 in humans [15]. It may also exert a similar effect on other organs that express these urate transporters [35]. Several early observations [36, 37] in patients with diabetes suggested that hyperglycemia may induce hypouricemia by increasing extracellular fluid volume and hence enhancing tubular UA excretion. In the present study, diabetic rats exhibited significantly lower SUA levels but higher UUA levels at Week 4. However, the significantly increased FEUA, rather than changes in Ccr, may suggest that decreased reabsorption of UA—rather than renal hyperfiltration—was responsible for the hypouricemia observed in these overtly hyperglycemic rats. Another reason for the decreased SUA in diabetic rats may be related to excess allantoin production, which can be attributed to the increased activation of hepatic uricase in these animals. Although mRNA expression in the liver was unaffected by either diabetes or BZ treatment, the activity of hepatic uricase and urinary allantoin levels were not assessed in the present study. However, several previous studies [38, 39], using urinary metabonomic analysis have, reported higher levels of allantoin in STZ-induced diabetic rats.

As we previously reported, BZ treatment in diabetic rats (DM + BZ group) significantly increased SUA levels, along with a further increase in urate excretion in the present experiment [40]. The uricosuric effect may directly result from the pharmacological action of BZ, but the increased SUA levels are different from the effects observed in humans after BZ treatment. It is well known that hepatic uricase in rodents plays a major role in maintaining UA homeostasis by converting it into water-soluble allantoin [41], and hepatic GLUT9 has been confirmed as the pivotal transporter in this physiological process [42]. To address this discrepancy, gene expressions related to UA metabolism in rat kidneys and livers were examined. We found that renal URAT1 expression was significantly inhibited, and GLUT9 expression remained unchanged, while hepatic GLUT9 expression was significantly inhibited, and the expression of uricase and XO were not affected after BZ treatment in diabetic rats. The inhibition of hepatic GLUT9 expression would decrease hepatic urate flow to the uricase pathway, leading to an elevation in SUA levels. This has been verified in several animal studies using liver-targeted GLUT9 knockout mice, which developed hyperuricemia and chronic renal injury [43–45]. Increased renal XO expression in diabetic rats, as well as increased hepatic uricase and XO expression in control rats after BZ treatment, may play only minor roles in SUA changes, but their true significance requires further research to clarify. Taking into account the results of gene expressions, we can deduce that BZ inhibits hepatic GLUT9 expression and increases SUA levels in diabetic rats, but adaptively promotes renal UA excretion through its potential inhibitory effects on renal URAT1 expression.

How does the inhibition of URAT1 benefit the progression of DKD in addition to its uricosuric effect? Several published studies [46, 47] have shown that the inhibition of URAT1 in the liver and adipose tissue may improve insulin resistance, lipid metabolism, tissue inflammation, and oxidative stress. In the present study, BZ treatment improved tubular hyaline degeneration to some extent, which may contribute to its renal protective effects, but the exact mechanisms still require further research to explore.

One limitation of the present study is that the STZ-induced rat model primarily mimics the pathophysiology of Type 1 diabetes, and these animals often progress rapidly to proteinuria and severe renal injury. In contrast, most patients with DKD have Type 2 diabetes. Although DKD arising from either Type 1 or Type 2 diabetes shares many clinical and histopathological features, growing evidence suggests that DKD in Type 2 diabetes more frequently exhibits glomerulomegaly, arteriolosclerosis, and tubulointerstitial fibrosis, changes driven in part by comorbid conditions such as hypertension and dyslipidemia [48]. We, therefore, hypothesize that BZ may also mitigate renal injury in Type 2 diabetes, but this will require further validation in models that better reflect the metabolic milieu of Type 2 diabetes (for example, obese rodents or low-dose STZ combined with a high-fat diet) and, ultimately, confirmation in clinical trials. Another limitation is the species-specific difference in uricase metabolism in mice, which may interfere with UA metabolism due to hepatic GLUT9 inhibition in rodents. Therefore, the use of an appropriate primate model will be necessary for future studies.

## 5. Conclusions

In this research, we found that BZ treatment significantly attenuated urinary albumin excretion and tubular hyaline degeneration in STZ-induced diabetic rats, independent of its influence on SUA and glucose levels. The renal protective effect may be attributed to its inhibition of renal URAT1 expression rather than GLUT9 expression. Additional basic and clinical research should be conducted to further elucidate the actual mechanisms involved.

## Figures and Tables

**Table 1:** Primer sequences for real time PCR.

**Rat gene**	**Primers (**5′-3′**)**
Actin	F: GGGAAATCGTGCGTGACATT R: GCGGCAGTGGCCATCTC
XO	F: TGATGGTTCGGTGCTGTTGAC R: GCTGTGGGAGAAGTGTTGGG
URAT1	F: GGTAGCAGCCCGAGGAGGAG R: TGGATGTCTTGGATGGTGTCAGG
GLUT9	F: TCCTACTGCTTCCTCGTCTTTGC R: ATGGGTTCTGTTCTTGGTCTCTGG
Uricase	F: CCTCTATGACATACAAGTGCTGAC R: GTGAATGTTTGGAAGGCTGATTTC

Abbreviations: F: forward sequence, R: reverse sequence.

## Data Availability

The original contributions presented in the study are included in the supplementary material. The genetic dataset analyzed during the current study are available in the Zenodo repository, with the persistent web link as following: 10.5281/zenodo.14500032.

## Nomenclature

ACR: albumin–creatinine ratio
ANOVA: one-way analysis of variance
BUN: blood urea nitrogen
BZ: benzbromarone
Ccr: clearance rate of creatinine
CKD: chronic kidney disease
CMC-Na: sodium carboxymethyl cellulose
DM: diabetes mellitus
DKD: diabetic kidney disease
eGFR: estimated glomerular filtration rate
ESRD: end-stage renal disease
FBG: fasting blood glucose
FEUA: fractional excretion of urate
GLUT9: glucose transporter 9
H&E: hematoxylin-eosin
HR: heart rate
LSD: least significant difference
MAP: mean arterial pressure
MGA: mean glomerular area
NC: normal control
RAS: renin–angiotensin system
SCr: serum creatinine
SUA: serum uric acid
STZ: streptozotocin
T2DM: Type 2 diabetes mellitus
UAE: urinary albumin excretion per day
ULT: uric acid-lowering therapy
UCr: urinary amounts of creatinine per day
UUA: urinary amounts of uric acid per day
UUN: urinary amounts of urea nitrogen per day
URAT1: urate transporter 1
XO: xanthine oxidase

## Supporting Information

Supporting Information Additional supporting information can be found online in the Supporting Information section. Table S1 Changes in body weight (g) among the groups. Table S2: Comparison of 24-h urine volume (mL) of rats in each group. Table S3: Changes in MAP and HR among the groups. Table S4: Comparison of fasting blood glucose in each group of rats. Table S5: Comparison of renal function among groups. Table S6: Comparison of SUA levels (*μ*mol/L) in rats among groups. Table S7: Comparison of 24-h UAE, UUA, UCR, and UUN in rats between groups. Table S8: Comparisons of mean glomerular area (MGA) and tubular hyaline degeneration score between groups.
