# Supplementary material for: Benzbromarone Inhibits Renal URAT1 and Attenuates Renal Damage in Streptozotocin-Induced Diabetic Rats, Independent of Its Uricosuric Effects
Source: J Diabetes Res. 2025 Nov 25;2025:9934190. doi: 10.1155/jdr/9934190 (PMC12672078; doi:10.1155/jdr/9934190)
Supplement: Supporting Information — Additional supporting information can be found online in the Supporting Information section. Table S1 Changes in body weight (g) among the groups. Table S2: Comparison of 24-h urine volume (mL) of rats in each group. Table S3: Changes in MAP and HR among the groups. Table S4: Comparison of fasting blood glucose in each group of rats. Table S5: Comparison of renal function among groups. Table S6: Comparison of SUA levels (μmol/L) in rats among groups. Table S7: Comparison of 24-h UAE, UUA, UCR, and UUN in rats between groups. Table S8: Comparisons of mean glomerular area (MGA) and tubular hyaline degeneration score between groups. [file 9934190.f1.docx]

**Table S1.** **Changes in body weight (g) among the groups.**

|  | NC | NC+BZ | DM | DM+BZ |
| --- | --- | --- | --- | --- |
| T0 | 352.88±24.75 | 345.65±18.08 | 228.01±16.45* | 233.75±19.82* |
| T4 | 502.63±42.93 | 486.75±38.64 | 258.79±34.42* | 259.58±28.96* |
| T8 | 526.66±63.98 | 518.63±49.43 | 202.91±98.70* | 198.16±27.69* |

NC: Normal control; BZ: Benzbromarone;

Data are expressed as mean ± standard deviation. T0, T4, and T8 indicate pre-intervention, 4 weeks and 8 weeks after intervention, respectively. *P<0.05 compared with the NC group.

**Table S2.** **Comparison of 24-hour urine volume (ml) of rats in each group.**

|  | NC | NC+BZ | DM | DM+BZ |
| --- | --- | --- | --- | --- |
| T0 | 10.92±2.75 | 12.25±2.67 | 129.09±31.62* | 124.58±34.49* |
| T4 | 12.42±6.33 | 13.42±4.98 | 147.55±56.08* | 129.50±34.04* |
| T8 | 10.17±6.74 | 14.25±3.70 | 139.45±25.17* | 115.25±36.32* |

NC: Normal control; BZ: Benzbromarone;

Data are expressed as mean ± standard deviation. T0, T4, and T8 indicate pre-intervention, 4 weeks and 8 weeks after intervention, respectively. *P<0.05 compared with the NC group.

**Table S3.** **Changes in MAP and HR among the groups.**

|  |  | NC | NC+BZ | DM | DM+BZ |
| --- | --- | --- | --- | --- | --- |
| MAP (mmHg) | T0 | 107±11 | 104±12 | 120±32 | 118±19 |
|  | T4 | 112±15 | 110±9 | 106±18 | 108±8 |
|  | T8 | 102±12 | 103±20 | 89±12 | 94±13 |
| HR (pulse/min) | T0 | 417±32 | 419±39 | 338±33* | 346±54* |
|  | T4 | 400±26 | 392±32 | 325±31* | 308±29* |
|  | T8 | 398±28 | 386±26 | 288±29* | 306±36* |

NC: Normal control; BZ: Benzbromarone; MAP: Mean Arterial Pressure; HR: Heart rate;

Data are expressed as mean ± standard deviation. T0, T4, and T8 indicate pre-intervention, 4 weeks and 8 weeks after intervention, respectively. *P<0.05 compared with the NC group.

**Table S4.** **Comparison of fasting blood glucose in each group of rats.**

|  |  | NC | NC+BZ | DM | DM+BZ |
| --- | --- | --- | --- | --- | --- |
| FBG (mmol/L) | T0 | 7.52±0.82 | 7.31±1.02 | 44.43±4.87* | 43.04±6.40* |
|  | T4 | 7.30±0.79 | 6.90±0.62 | 35.15±5.50* | 32.36±2.90* |
|  | T8 | 5.56±0.54 | 5.75±0.63 | 33.00±11.61* | 32.66±4.23* |

NC: Normal control; BZ: Benzbromarone; FBG: Fasting blood glucose;

Data are expressed as mean ± standard deviation. T0, T4, and T8 indicate pre-intervention, 4 weeks and 8 weeks after intervention, respectively. *P<0.05 compared with the NC group.

**Table S5.** **Comparison of renal function among groups.**

|  |  | NC | NC+BZ | DM | DM+BZ |
| --- | --- | --- | --- | --- | --- |
| SCr (μmol/L) | T0 | 24.64±2.29 | 25.89±2.36 | 28.07±2.49 | 24.74±4.13 |
|  | T4 | 23.58±1.70 | 25.89±3.76 | 25.72±14.35 | 20.61±4.25 |
|  | T8 | 30.35±4.75 | 33.99±8.18 | 25.42±9.65* | 24.85±3.60* |
| BUN (mmol/L) | T0 | 5.64±0.53 | 5.41±0.42 | 10.40±2.17* | 10.04±1.20* |
|  | T4 | 5.08±0.43 | 5.17±0.95 | 12.77±2.91* | 11.11±2.74* |
|  | T8 | 6.68±1.03 | 6.82±1.21 | 10.53±4.41* | 13.10±3.26* |

NC: Normal control; BZ: Benzbromarone; SCr: Serum creatinine; BUN: Blood urea nitrogen;

Data are expressed as mean ± standard deviation. T0, T4, and T8 indicate pre-intervention, 4 weeks and 8 weeks after intervention, respectively. *P<0.05 compared with the NC group.

**Table S6.** **Comparison of SUA levels (μmol/L) in rats among groups.**

|  | NC | NC+BZ | DM | DM+BZ |
| --- | --- | --- | --- | --- |
| T0 | 80.74±11.61 | 78.44±10.73 | 67.32±13.47* | 71.94±17.93* |
| T4 | 76.01±8.81 | 80.72±16.49 | 52.97±9.84* | 79.71±27.67# |
| T8 | 85.78±15.94 | 91.08±21.14 | 66.47±12.73* | 93.84±15.80# |

NC: Normal control; BZ: Benzbromarone; SUA: Serum uric acid;

Data are expressed as mean ± standard deviation. T0, T4, and T8 indicate pre-intervention, 4 weeks and 8 weeks after intervention, respectively. *P<0.05 compared with the NC group, # P<0.05 compared with the DM group.

**Table S7.** **Comparison of 24-hour UAE, UUA, UCR, and UUN in rats between groups.**

|  |  | NC | NC+BZ | DM | DM+BZ |
| --- | --- | --- | --- | --- | --- |
| UAE (μmol/24h) | T0 | 39.93±14.96 | 44.11±11.64 | 765.26±155.76* | 743.77±208.15* |
|  | T4 | 38.45±22.60 | 40.17±15.97 | 857.47±369.57* | 653.60±193.07*# |
|  | T8 | 32.65±23.10 | 41.57±11.58 | 776.49±278.10* | 577.66±195.89*# |
| UUA (μmol/24h) | T0 | 11.67±3.82 | 10.64±3.62 | 12.41±5.24 | 11.83±3.14 |
|  | T4 | 11.90±5.38 | 12.29±4.37 | 14.32±6.18*# | 16.08±5.67* |
|  | T8 | 8.59±4.09 | 11.11±3.14 | 15.27±6.87 | 15.22±7.67* |
| UCr (μmol/24h) | T0 | 75.20±16.49 | 65.53±15.78 | 86.13±68.81 | 91.76±58.36 |
|  | T4 | 76.96±33.71 | 60.99±27.03 | 67.66±32.48 | 66.25±14.48 |
|  | T8 | 57.47±24.57 | 79.81±21.41 | 65.51±32.75 | 57.54±22.44 |
| UUN (mmol/24h) | T0 | 1.02±0.24 | 1.06±0.26 | 12.70±3.73* | 13.87±4.11* |
|  | T4 | 0.81±0.59 | 1.02±0.65 | 12.69±5.00* | 11.55±3.33* |
|  | T8 | 0.72±0.65 | 0.94±0.51 | 10.50±3.81* | 9.80±3.62* |
| Ccr (ml/min) | T0 | 2.12±0.44 | 1.77±0.44 | 2.13±1.82 | 2.53±1.47 |
|  | T4 | 2.28±1.01 | 1.66±0.70 | 2.17±1.16 | 2.31±0.62 |
|  | T8 | 1.32±0.54 | 1.71±0.58 | 1.83±0.72 | 1.61±0.59 |
| FEUA (%) | T0 | 4.82±1.53 | 5.34±0.86 | 11.03±7.55* | 12.75±6.73* |
|  | T4 | 5.02±1.44 | 10.33±9.44 | 8.84±3.38 | 7.10±3.93 |
|  | T8 | 5.44±1.66 | 5.30±1.26 | 9.27±5.24* | 7.83±2.41* |

NC: Normal control; BZ: Benzbromarone; UAE: Urinary albumin excretion; UUA: Urinary uric acid; UCR: Urinary creatinine; UUN: Urinary urea nitrogen; Ccr: Clearance rate of creatinine; FEUA: Fractional excretion of urate.

Data are expressed as mean ± standard deviation. T0, T4, and T8 indicate pre-intervention, 4 weeks and 8 weeks after intervention, respectively. *P<0.05 compared with the NC group, # P<0.05 compared with the DM group.

**Table S8.** **Comparisons of mean glomerular area (MGA) and tubular hyaline degeneration score between groups**

|  | NC | NC+BZ | DM | DM+BZ |
| --- | --- | --- | --- | --- |
| MGA  (μm^2^) | 9093.73±739.81 | 8897.220±571.08 | 8222.96±699.75* | 8467.72±755.76 |
| Tubular hyaline degeneration score | 0.17±0.39 | 0.25±0.45 | 1.42±0.79* | 0.92±0.52*# |

Data are expressed as mean ± standard deviation. *P<0.05 compared with the NC group, # P<0.05 compared with the DM group.
